# Use of melatonin in mild cognitive impairment: a retrospective study

**DOI:** 10.1002/alz.093299

**Published:** 2025-01-03

**Authors:** Natividad Olivar, Luis Ignacio Brusco, Daniel Pedro Cardinali

**Affiliations:** ^1^ University of Buenos Aires, Buenos Aires Argentina; ^2^ ALZAR ‐ Argentine Alzheimer’s Association, Buenos Aires, CABA Argentina; ^3^ National Scientific and Technical Research Council (CONICET), Buenos Aires Argentina

## Abstract

**Background:**

Variations in the circadian rhythm in older adults can negatively affect brain and cardiovascular health. As a consequence, poor cognitive functioning, an increase in the inflammatory response, alterations in sleep patterns, changes in mood and a decrease in motivation, energy and initiative are observed.

**Method:**

We retrospectively examined the initial and final neuropsychological assessment of 250 MCI outpatients, 125 of whom had received daily 3‐9 mg of a fast‐release melatonin preparation p.o. at bedtime for 12 months. Melatonin was given in addition to the standard medication prescribed by the attending psychiatrist.

**Result:**

Patients treated with melatonin showed significantly better performance in Mini Mental State Examination and the cognitive subscale of the Alzheimer’s Disease Assessment Scale. After application of a battery of neuropsychological tests including Mattis test, Digit‐symbol test, Trail A and B tasks and the Rey’s verbal test, better performance was found in melatonin‐treated patients, except for the Digit‐symbol test score which remained unchanged. Abnormally high Beck Depression Inventory scores decreased in melatonin‐treated patients, concomitantly with an improvement in wakefulness and sleep quality.

**Conclusion:**

The results suggest that melatonin can be a useful add‐on drug for treating MCI in a clinical setting.

